# Closure of atrial septal defect normalizes left and right ventricular hemodynamic forces in children and adolescents

**DOI:** 10.1016/j.jocmr.2026.102704

**Published:** 2026-02-12

**Authors:** Per M. Arvidsson, Henning Clausen, Pia Sjöberg

**Affiliations:** a Clinical Physiology, Department of Clinical Sciences Lund, Lund University, Lund, Sweden; b Department of Clinical Physiology, Skåne University Hospital, Lund, Sweden; c Pediatric Cardiology, Department of Clinical Sciences Lund, Lund University, Lund, Sweden; d Department of Pediatric Cardiology, Children’s Heart Center, Skåne University Hospital, Lund, Sweden

**Keywords:** Atrial septal defect, 4D flow, Hemodynamic force analysis, Intervention, Congenital heart disease

## Abstract

**Background:**

Atrial septal defect (ASD) is one of the most common congenital heart defects and leads to chronic right ventricular (RV) volume loading, which may progress to heart failure if untreated. Surgical or transcatheter closure unloads the RV and improves hemodynamics, but the impact on intraventricular hemodynamic forces (HDF) remains unclear. Here, we evaluated the effects of ASD closure on biventricular HDF.

**Methods:**

Cardiac magnetic resonance imaging was performed at 1.5T on 21 patients and 11 sex- and age-matched healthy controls. Whole-heart 4D flow and cine images in standard views were acquired and used to compute HDF using custom software (Segment). Paired analysis was used to compare HDF pre-/post ASD closure.

**Results:**

At baseline, patients showed aberrant RV HDF in both longitudinal and transverse directions (p<0.05 vs controls), largely explained by volume loading (R = 0.83–0.92 for volume parameters, p<0.0001). At 8-month follow-up (n = 16), LV HDF increased in the apical-basal and septal-lateral directions (p<0.05), while RV HDF decreased in all directions (p<0.05), effectively normalizing HDF to control levels (p>0.05). Changes in HDF were associated with remodeling of ventricular volumes and with shunt reduction.

**Conclusion:**

Children and adolescents with ASD exhibit a wide range of alterations to ventricular HDF partially reflective of altered loading conditions. Successful ASD closure normalizes HDF in many but not all cases, highlighting the potential of force analysis to improve our understanding of ventricular remodeling processes in congenital heart disease.

## Graphical abstract

**Figure:**
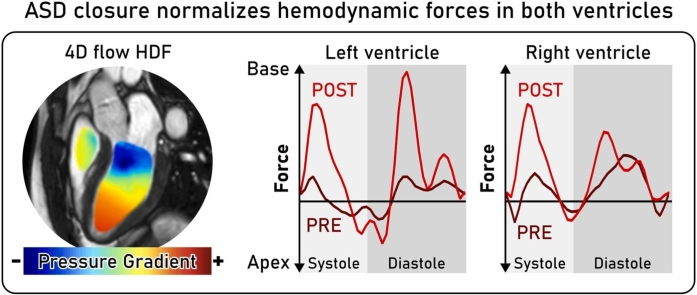

## 1 Background

Atrial septal defect (ASD) is one of the most common congenital heart defects, with an estimated prevalence approximating 1/1000 births [1]. In most patients, an ASD results in a left-to-right shunt, the magnitude of which is governed by the defect dimensions and the interatrial pressure gradient [2]. While many patients with small shunts experience little or no symptoms, larger shunts will expose the right ventricle (RV) and the pulmonary circulation to significant volume loading. This increase in RV preload causes flattening of the interventricular septum, leading to left ventricular (LV) underfilling and hence diminished systemic cardiac output. Untreated, a hemodynamically significant ASD may lead to gradual dilatation and hypertrophic remodeling of the right heart [3], replacement fibrosis, pulmonary hypertension, and in some patients, heart failure [4].

Treatment of ASD involves either transcatheter occlusion or surgical closure, where early intervention is generally favored when possible [5]. Following ASD closure, patients have a long-term risk profile largely similar to that of the general population, [6], [7], [8] and a number of studies have demonstrated improvement in LV dimensions and function after ASD closure in children and young adults. However, for reasons largely unknown, exercise capacity as well as left ventricular volumes and function may remain depressed for decades after surgery [5], [8], [9], [10], [11]. Recent studies have suggested that underfilling may cause a relatively underdeveloped LV through mechanotransductory pathways, [12], [13] leading to increased myocardial stiffness as well as impaired diastolic function through decreased hydraulic forces in early diastole [14]. In some cases, an underdeveloped LV may be insufficiently compliant to compensate for the sudden preload increase that follows a successful ASD closure, contributing to a state with impaired cardiac output and/or pulmonary edema. [15].

According to current guidelines, ASD closure is recommended in all patients with unequivocal evidence of RV overload with no signs of pulmonary arterial hypertension or significant left ventricular disease, regardless of the symptom burden [16]. However, in patients with LV disease, the benefit of eliminating the left-right shunt must be carefully weighed against unwanted outcomes resulting from a post-operatively overloaded LV [16]. Furthermore, reliable echocardiographic imaging of the right ventricle is hampered by its complex shape and position. And as many patients have grown accustomed to the functional impairment, correctly interpreting the clinical significance of a subtly remodeled right ventricle can be challenging. Better imaging markers are therefore needed to monitor biventricular function, improve surgical planning, and assess the hemodynamic effects of ASD closure.

Hemodynamic force (HDF) analysis from four-dimensional (4D) flow cardiovascular magnetic resonance (CMR) is one such emerging imaging biomarker of cardiac function [17], [18], [19], [20]. Hemodynamic forces describe the directional and temporal distribution of forces involved in ventricular blood acceleration, and hence the instantaneous exchange of forces between the blood and surrounding myocardium [21], [22], [23]. HDF analysis has been suggested to convey unique information about mechanisms driving remodeling processes and may complement traditional volumetric and functional imaging metrics [22]. For example, one study used HDF analysis to assess whether pulmonary valve replacement normalizes the hemodynamic conditions in Tetralogy of Fallot patients [24].

As a first step toward understanding how altered loading in ASD affects intraventricular force patterns, in the present exploratory study, we sought to evaluate whether pediatric ASD patients display aberrant HDF compared to healthy controls, and whether ASD closure normalizes these forces.

## 2 Methods

The study was approved by the Swedish Ethical Review Authority (2019–05490) and was conducted in accordance with the Helsinki Declaration. Written informed consent was obtained from the participants’ parents after considering the child’s wish to participate. The study is reported in accordance with the STROBE guidelines [25].

This prospective, longitudinal case-control study was conducted at a Swedish tertiary care center for congenital heart defects (ClinicalTrials.gov ID: NCT04667455). Patient inclusion was from March 2021 to February 2023. Twenty-two children under the age of 18 years with ASD, with or without partially anomalous venous drainage, referred and accepted for ASD closure were prospectively and non-randomly recruited based on the chronological order of planned intervention.

ASD closure was performed according to standard clinical practice. Patients with suitable defect anatomy underwent transcatheter closure via femoral venous access using a septal occluder device. Patients not eligible for transcatheter intervention underwent surgical closure with direct suture closure or patch repair under cardiopulmonary bypass, with concurrent correction of partial anomalous pulmonary venous return if present. The choice of closure technique was based on anatomical considerations and made independently of the imaging study.

Exclusion criteria were contraindication to CMR or need for general anesthesia to undergo CMR. Patients underwent CMR examination within 24 h before intervention and 6–12 months after closure. While no patients were examined under general anesthesia, sedation by dexmedetomidine at 2–3 µg/kg/dose intranasally (Dexdor, Orion Pharma, Espoo, Finland) was used per clinical routine.

Healthy pediatric volunteers were also examined to provide a control group. Controls were recruited with the intention to achieve 1:2 matching for age and sex to patients on the group level. Controls had normal ECG and were free from medication, known cardiovascular disease, and hypertension.

### 2.1 CMR imaging

Participants underwent CMR at 1.5T (Aera, Siemens Healthineers, Erlangen, Germany) with a cardiac coil. The imaging protocol included balanced steady-state free precession (bSSFP) cine images in the standard short-axis and long-axis views, as well as three-dimensional, time-resolved phase contrast (4D flow) images with coverage of the entire heart. Retrospective ECG gating was used for both bSSFP and flow imaging.

A 4D flow imaging was performed using a validated prototype sequence with Cartesian readout [26], with a sample volume covering the heart and proximal great vessels. Respiratory navigator gating was employed using 90°–180° cross-pair navigator pulses centered over the right liver lobe. Typical imaging parameters for 4D flow: echo time/repetition time: 3.5/5.7 ms, flip angle 8°, VENC 150 cm/s, acquired temporal resolution 45 ms reconstructed to 25 time phases, matrix size 96×80×52, spatial resolution 2.2×2.2×2.5 mm^3^, partial Fourier factor 0.75 in phase and slice directions, and no slice gap. Parallel imaging was used to accelerate data acquisition (GRAPPA factor 2 in anterior-posterior phase encode direction and 2 in superior-inferior slice encode direction) [27], and temporal segmentation factor 2.

### 2.2 Image analysis

All image analysis was performed by one reader (PMA, 15 years CMR experience) using Segment 4.0 R12067 (Medviso, Lund, Sweden) with publicly available, in-house developed plugins [28]. Biventricular volumes were delineated over the cardiac cycle using semi-automated detection of the endocardial borders, with manual adjustments as needed. Ventricular volumes were indexed to body surface area (BSA, calculated using Mosteller’s formula) for all between-group and longitudinal comparisons.

Quality control steps for the 4D flow datasets included visual assessment of data quality for each individual phase encoding direction as well as the magnitude images. Residual phase background errors were automatically detected and corrected using fitting to stationary tissue [29] and aliasing errors were corrected using automatic phase unwrapping [30]. The spatial alignment of the cine images and delineations was visually assessed and manually corrected to align with the 4D flow data.

**Fig. 1:**
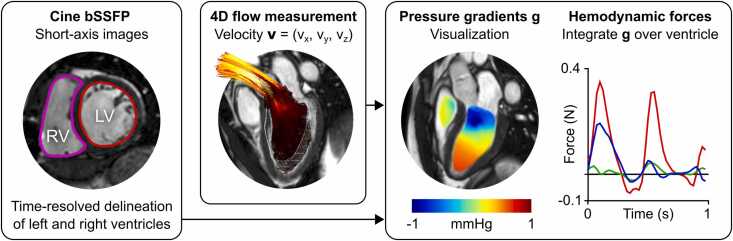
Method overview for hemodynamic force analysis. Left, cine loops are acquired in the short-axis views and biventricular volumes contoured over the full cardiac cycle. Center, four-dimensional flow is acquired with coverage of both ventricles. Right, pressure gradients may be computed using the Navier-Stokes equations and visualized as a time-varying relative pressure field. Integrating the sum of all regional pressure gradients over the entirety of the ventricle produces an instantaneous global hemodynamic force, which may then be decomposed into three orthogonal components for reproducible quantification

Hemodynamic forces were quantified for the left and right ventricles separately using a validated method previously described in greater detail [17], [18]. An overview is provided in Fig. 1. Briefly, intraventricular pressure gradients were computed from the 4D flow dataset using the Navier-Stokes equation and then integrated over the ventricular volume for each point in time, producing a time-varying global force vector for each ventricle.

This vector was then decomposed into its three orthogonal components as previously described [17]. First, the end-diastolic position of the atrioventricular valve plane (AV plane) was manually determined in the long-axis images. This plane was then used as a spatial reference for the apical-basal force component, orthogonal to the AV plane. The apical-basal component captures the main inflow-outflow axis of blood transit through the left ventricle and normally dominates in the LV in both systole and diastole [17], [31]. The lateral-septal component was orthogonal to the apical-basal component and aligned with the left ventricular outflow tract, and thus captures the transverse acceleration associated with redirection of flow during systole, as well as with septal motion patterns. Finally, the inferior-anterior component was orthogonal to the other two, and normally contains little signal.

For the right ventricle (RV), the same spatial reference directions were used, although the axes were renamed to reflect the differing anatomical landmarks (septum-free wall, diaphragm-RVOT). The RV geometry results in a more prominent role of transverse forces, with the diaphragm-RVOT direction reflecting the inflow–outflow pathway while the septum-free wall captures the curved redirection of flow along the septum [32] as well as the septal contribution to RV ejection [17].

To facilitate comparison between subjects with varying resting heart rates, HDF curves were resampled to a reference cardiac cycle using linear interpolation with end-systole set at 40% of the R-R interval [20]. Peak and root mean square (RMS) HDF values were calculated for each directional component in systole and diastole, as previously described [18].

### 2.3 Statistical methods

Due to the limited sample size and paucity of published data regarding how hemodynamic forces are distributed, each HDF parameter was first tested for normality using the Shapiro-Wilk test and Q-Q plots. For normally or lognormally distributed parameters, inter- and intragroup comparisons were made using the paired and unpaired t-test, respectively. Associations were assessed with Pearson correlation and visualized using linear regression. Lognormal parameters were log-transformed for analysis but are presented in their original scale to facilitate interpretation.

Non-normally distributed parameters were compared between groups using the Mann-Whitney U test, and paired comparisons were performed using the Wilcoxon signed-rank test. Associations involving non-normally distributed parameters were tested using Spearman correlation and visualized with linear regression for descriptive purposes. Categorical variables were evaluated using the chi-square test.

Numerical data are given as median [interquartile range], unless otherwise stated. Statistical significance was assigned at p<0.05. As this was a hypothesis-generating study, we did not correct for multiple testing. Due to the paucity of preexisting data in the literature, and the explorative nature of the study, we did not perform a power calculation.

## 3 Results

**Fig. 2:**
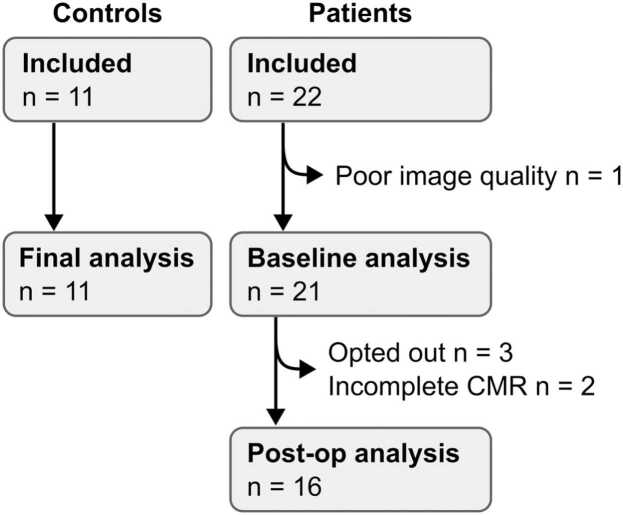
Inclusion flowchart

Fig. 2 shows the inclusion flow chart. Twenty-two children underwent CMR before ASD closure. Eleven healthy controls also underwent CMR examination. The overall CMR examination was 43 ± 12 min (mean ± SD), of which 4D flow acquisition was 8 ± 3 min. Three patients opted out after the procedure, one study had insufficient image quality, and two patients were unable to complete the 4D flow scan despite sedation. Baseline analysis was therefore conducted in 11 controls and 21 patients, while complete datasets of adequate quality before and after ASD closure were available in 16 patients.

### 3.1 Baseline data

Table 1 summarizes the subject characteristics. Controls and patients were well matched at the group level for age and sex. Further, controls and patients had similar height, weight, body surface area, biventricular ejection fraction, and resting heart rate (p>0.05 for all).

**Table 1:** Subject characteristics.

	Controls (n = 11)	P (control vs preop)*	ASD preop (n = 21)	P (pre vs postop)†	ASD postop (n = 16)	P (control vs postop)*
Age, y	10 [7–13]	0.71	8 [5–13]		10 [6–14]	0.67
Sex	6 m, 5 f	0.15	6 m, 15 f	0.57	5 m, 11 f	0.23
Height, cm	142 [128–168]	0.36	138 [110–161]	**<0.0001**	153 [116–166]	0.91
Weight, kg	32 [23–43]	0.74	30 [20–51]	**0.0005**	38 [23–61]	0.25
BSA, m^2^	1.12 [0.90–1.42]	0.73	1.07 [0.78–1.48]	**0.0002**	1.34 [0.86–1.66]	0.41
Resting heart rate, bpm	75 [67–84]	0.10	83 [75–92]	0.14	77 [62–97]	0.75
LV EDVi, mL/m^2^	83 [78–93]	**<0.0001**	63 [58–70]	**0.0002**	76 [71–87]	0.10
LV SVi, mL/m^2^	49 [46–54]	**<0.0001**	39 [33–43]	**0.04**	47 [39–52]	0.27
LV EF, %	57 [54–63]	0.53	60 [56–64]	0.27	59 [51–65]	0.94
Cardiac index, l/min/m^2^	3.7 [3.3-4.3]	**0.02**	3.4 [2.8–3.6]	0.13	3.4 [2.9–4.1]	0.16
RV EDVi, mL/m^2^	88 [80–95]	**<0.0001**	129 [110–152]	**<0.0001**	90 [81–106]	0.83
RV SVi, mL/m^2^	50 [45–55]	**<0.0001**	72 [61–91]	**<0.0001**	49 [37–54]	0.48
RV EF, %	57 [52–61]	0.53	58 [55–62]	**0.001**	49 [44–59]	0.056
Planimetric Qp/Qs	1.03 [0.96–1.06]	**<0.0001**	1.79 [1.53–2.6]	**<0.0001**	1.04 [0.98–1.09]	0.39

Data are medians [interquartile range]. Bold indicates values of statistical significance.

*ASD* Atrial septal defect, *BSA* body surface area, *LV* left ventricular, *EDV* end-diastolic volume, *EF* ejection fraction, *RV* right ventricular, *SVi* stroke volume index, *Qp/Qs* ratio of pulmonic to systemic flow.

* Mann-Whitney U test

† Wilcoxon signed-rank test, except for sex distribution, which was evaluated using the chi-square test

Compared to controls, patients had significantly smaller LV volumes and larger RV volumes at baseline, owing to significant left-to-right shunting with a median Qp/Qs of 1.79 (range: 1.24–3.73). Among the included patients, one had a superior sinus venosus defect with right-sided PAPVR, which was treated surgically. All remaining patients had secundum-type defects, including one additional PAPVR case.

### 3.2 Intervention and follow-up

Of the 16 patients who completed both baseline and follow-up examinations, 13 underwent catheter closure, and 3 had open-chest surgery. Surgery was performed the day after baseline CMR in all except two cases, where attempts at catheter closure were unsuccessful due to difficulty in attaining a good fit for the device. In these cases open surgery was performed 147 and 182 days after the baseline CMR, respectively. Closure was successful in all cases, as evidenced by normalization of Qp/Qs at the follow-up CMR after a median of 8.2 months (range: 5.5–11 months). In the intervening time, patients increased in height by a median of 3 centimeters, weight by a median of 4 kg, and BSA by 0.08 m^2^. To compensate for changes in body dimensions, we consider BSA-indexed values for before-after comparisons of cardiac volumetry.

### 3.3 Postoperative ventricular remodeling

After intervention, patients’ LV end-diastolic volume index (EDVi) had increased significantly (Table 1) and was no different from controls (p = 0.10), reflecting the improved ventricular filling resulting from reduced shunting. This was accompanied by increased LV stroke volume index (SVi), to being no different to controls (p = 0.27), while LV EF and cardiac index were unaffected.

Meanwhile, RV EDVi decreased significantly to being no different from controls (p = 0.83), as did RV SVi (p = 0.48). The RV EF also decreased significantly but was not significantly lower than in controls (p = 0.056).

### 3.4 Hemodynamic forces at baseline and after ASD closure

**Fig. 3:**
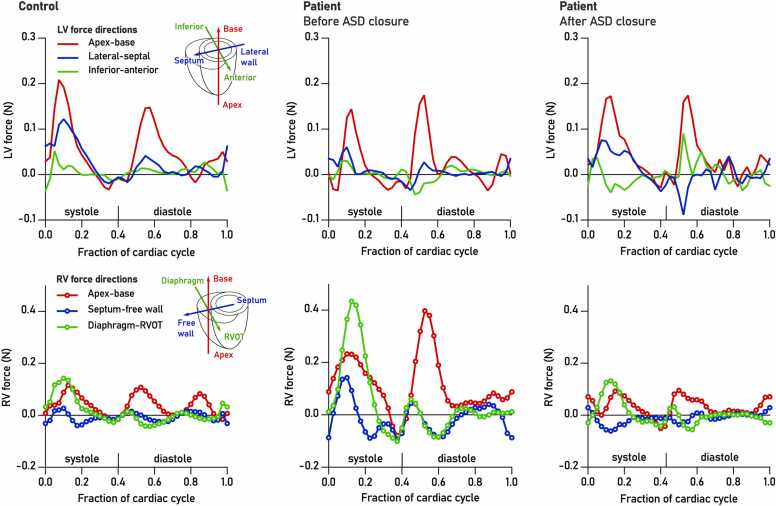
Example of hemodynamic forces (HDF) in control (left), patient before ASD closure (center), and patient after surgery (right). Top row: left ventricular HDF, bottom row: right ventricular HDF. Different curve rendering styles are used to facilitate visual separation of LV and RV force traces. Note the relatively unaltered LV forces after closure (top right), and the changes to both systolic and diastolic forces in the RV, indicating both altered loading conditions (red and green dotted lines) and their effects on the role of the interventricular septum (blue dotted line). *ASD* atrial septal defect, *HDF* hemodynamic forces, *LV* left ventricular, *RV* left ventricular

Fig. 3 shows examples of LV and RV HDF in one control and one patient. In this patient, LV HDF was similar to the control, while RV forces were markedly increased. Closure normalized RV HDF in both systole and diastole.

**Fig. 4:**
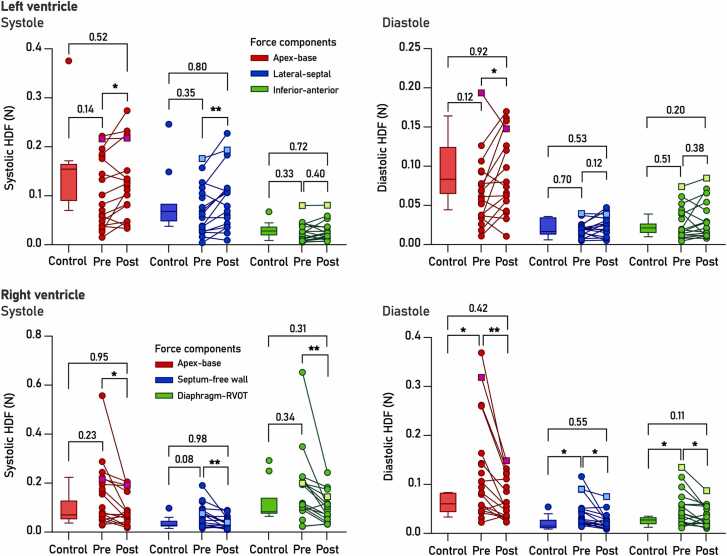
Biventricular HDF in controls and in patients before (Pre) and after (Post) ASD closure. Top, left ventricular HDF; bottom, right ventricular HDF. RMS (root mean square) values shown for systole and diastole separately. Squares denote a selected individual with persistently elevated HDF post closure, discussed separately. Controls shown as Tukey plots for visual clarity. Note different scales on Y axes. *, p<0.05; **, p<0.01. *ASD* atrial septal defect, *HDF* hemodynamic forces, *RMS* root mean square

Fig. 4 shows biventricular HDF in controls and patients before and after ASD closure (RMS values for systole and diastole separately). Numerical data are shown in Table 2. Supplemental figure 1 shows biventricular HDF curves (average values ±SD) for controls and patients.

**Table 2:** Hemodynamic forces in controls and patients before and after ASD closure.

	Controls (n = 11)	P (control vs preop)*	ASD preop (n = 21)	P (pre vs postop)†	ASD postop (n = 16)	P (control vs postop)*
*LV HDF – Systole*						
Apex-base	0.147	0.14	0.104	**0.04**	0.127	0.52
Lateral-septal	0.086	0.35	0.067	**<0.01**	0.092	0.80
Inferior-anterior	0.031	0.33	0.024	0.40	0.028	0.72
*LV HDF – Diastole*						
Apex-base	0.091	0.12	0.067	**0.04**	0.089	0.92
Lateral-septal	0.020	0.70	0.019	0.12	0.023	0.53
Inferior-anterior	0.022	0.51	0.026	0.38	0.031	0.20
*RV HDF – Systole*						
Apex-base	0.097	0.23	0.146	**0.03**	0.095	0.95
Septum-free wall	0.038	0.08	0.064	**<0.01**	0.038	0.98
Diaphragm-RVOT	0.125	0.34	0.169	**<0.01**	0.098	0.31
*RV HDF – Diastole*						
Apex-base	0.060	**0.04**	0.128	**<0.01**	0.071	0.42
Septum-free wall	0.022	**0.05**	0.041	**0.02**	0.026	0.55
Diaphragm-RVOT	0.026	**0.02**	0.050	**0.02**	0.037	0.11

Data are mean RMS values expressed in Newton (N). Bold indicates values of statistical significance.

* unpaired T test

† paired T test

### 3.5 Left ventricular HDF

In the LV, neither systolic nor diastolic HDF differed between controls and patients before closure (Student’s t p>0.1 for all).

Following ASD closure, systolic LV HDF increased significantly in the apex-base and lateral-septal directions, suggestive of increased LV stroke work, yet remained similar to controls at the group level (p>0.5 for both).

For diastole, apex-base HDF increased significantly (p<0.05) to normal levels (p = 0.92 vs controls) after closure, indicative of improved LV filling. Lateral-septal and inferior-anterior HDF did not change significantly following closure (p>0.1 for both), and remained comparable to controls (p>0.2 for all).

### 3.6 Right ventricular HDF

In systole, RV HDF did not differ significantly between controls and ASD patients before closure (Fig. 4, bottom row, p>0.05 for all), although individual patients frequently displayed higher HDF in the septum-free wall direction. In diastole, patients displayed significantly elevated forces in the apex-base, diaphragm-RVOT, and septum-free wall directions compared to controls (p<0.05 for all).

ASD closure brought about significantly lower systolic forces in all three directions (p<0.05 for all), bringing the patients closer to values seen in controls. Diastolic forces also decreased significantly (p<0.05 for all) to the point of being similar to controls (p>0.1 for all).

In the patient with the highest RV HDF at follow-up (marked with squares in Fig. 4), RV HDF was also among the highest at baseline. This patient underwent ASD closure at 17 years of age and exhibited larger absolute ventricular dimensions (RV EDV 300 mL) compared with the cohort median (94 mL). Following closure, Qp/Qs decreased from 1.8 to 1.0, and RV EDVi decreased from 133 to 107 mL/m² (control median: 85 mL/m^2^), while RV HDF remained higher than in controls at follow-up.

### 3.7 Associations with baseline HDF

**Fig. 5:**
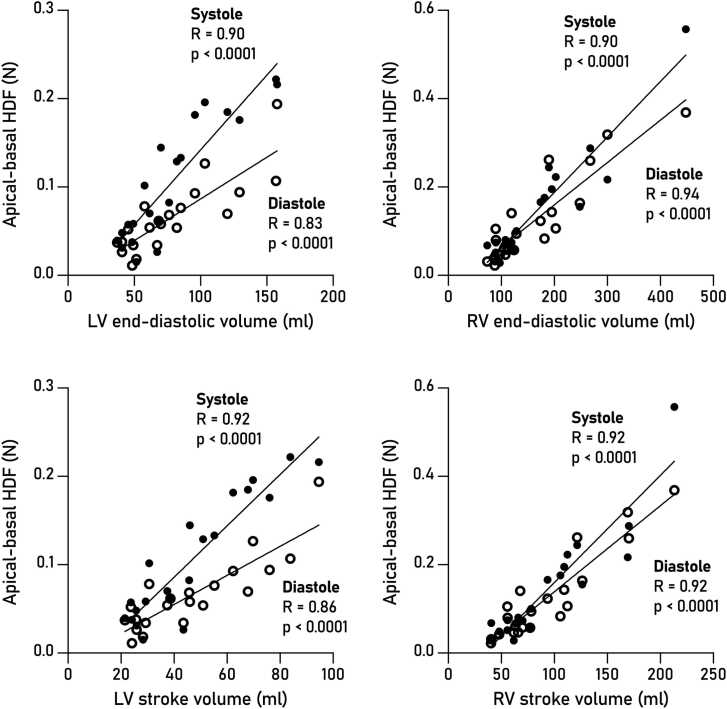
Pearson correlations of volumetric parameters associated with longitudinal HDF at baseline, with RMS values for systole and diastole separately. Pearson R and p values from non-log transformed data. Filled circles, systolic HDF; open circles, diastolic HDF. *HDF* hemodynamic forces, *RMS* root mean square

Biventricular HDF before ASD closure was associated with height, weight, and BSA (Pearson R = 0.58–0.88, p<0.05), reflecting that larger individuals have larger hearts with greater forces. More specifically, Fig. 5 shows that LV HDF were associated with LV end-diastolic volume (R = 0.71–0.92 for systole, 0.63–0.77 for diastole, p<0.05), and with LV stroke volume (R = 0.76–0.93 for systole, 0.68–0.88 for diastole, p<0.05). Right ventricular HDF were similarly associated with RV end-diastolic volume (Pearson R = 0.70–0.75 for systole, 0.52–0.65 for diastole, p<0.05) and with RV stroke volume (R = 0.71–0.80 for systole, 0.53–0.63 for diastole, p<0.05). Hemodynamic forces were not associated with ejection fraction nor with shunt fraction (Qp/Qs), p>0.05 for all.

### 3.8 Changes in HDF following ASD closure

**Fig. 6:**
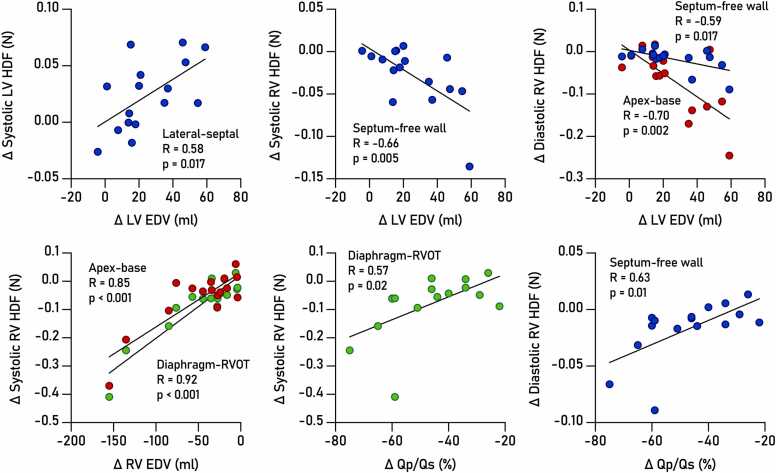
Linear regression analysis of parameters significantly associated with changes in HDF after ASD closure. Top row: Increasing LV end-diastolic volume was significantly associated with increased systolic LV HDF in the lateral-septal direction, and conversely with decreased systolic RV HDF in the septum-free wall direction. EDV augmentation was also inversely correlated with diastolic RV HDF. Bottom row: Decreasing RV end-diastolic volume was associated with decreased systolic RV HDF in the apex-base and diaphragm-RVOT directions. Furthermore, RV HDF was also associated with reductions in shunt fraction (ΔQp/Qs). *ASD* atrial septal defect, *HDF* hemodynamic forces, *LV* left ventricular, *RV* left ventricular, *EDV* end-diastolic volume

For the left ventricle, a positive correlation was seen between changes in end-diastolic volume and systolic HDF in the septal-lateral direction (Spearman R = 0.57, p<0.05), indicating a coupling between improved LV filling and altered septal dynamics. Further, we observed negative correlations between changes in RV HDF and left ventricular volumes (Spearman R = −0.51 to −0.64, p<0.05), indicating unloading of the right ventricle coupled to improved LV pumping. Meanwhile, changes in RV HDF were positively correlated with changes in RV volumes (Spearman R = 0.52–0.82, p<0.05) as well as with the % reduction of shunt fraction (Spearman R = 0.54–0.62, p<0.05). Changes in ejection fraction were not associated with changes in HDF. Fig. 6 shows linear regressions for selected associations.

## 4 Discussion

In this manuscript, we present an analysis of hemodynamic forces from 4D flow CMR in a cohort of children and adolescents before and after undergoing atrial septal defect closure. Compared to age- and sex-matched healthy controls, the patients exhibited significantly disturbed hemodynamic forces, most markedly in the right ventricle. After closure, reduced shunting and hence improved biventricular loading conditions largely restored HDF to levels seen in controls. Hemodynamic force analysis may complement existing approaches for assessing the hemodynamic consequences of load-altering interventions such as ASD closure.

### 4.1 Hemodynamic forces in relation to ventricular volume loading

While ventricular volumes showed strong associations with HDF, this should not be interpreted as a direct equivalence. Hemodynamic forces reflect the time derivative of intraventricular momentum, and the net force exchanged between the myocardium and intraventricular blood is therefore governed by the effective mass of blood undergoing coherent acceleration, and the associated acceleration itself, in accordance with Newton’s second law (F = m·a). Importantly, the mass term does not represent total ventricular blood volume as a lumped quantity, but rather the portion of blood that participates in organized, time-varying motion contributing to net momentum change.

In the setting of a volume-loaded yet mechanically functioning ventricle, such as in ASD, intraventricular flow organization is expected to be relatively preserved. Under these conditions, increases in chamber size are accompanied by increases in the effective accelerating blood mass, explaining why HDF was strongly correlated with end-diastolic and stroke volumes and changed predictably with loading conditions, as suggested in previous studies of healthy subjects and athletes [17], [31].

This contrasts with pathological states characterized by regional or global ventricular dysfunction, where blood flow patterns are more heterogeneous and substantial portions of ventricular blood volume may contribute little to net momentum exchange. In such settings, increased chamber volume does not necessarily translate into increased HDF. For example, in a previous study on heart failure patients with varying degrees of systolic dysfunction, HDF were similar to controls despite significantly increased ventricular volumes in some groups [19]. Regional functional abnormalities such as ventricular dyssynchrony may therefore profoundly affect the distribution and magnitude of HDF independent of ventricular blood mass, as demonstrated previously [20], [33].

Consequently, changes in ventricular loading constitute a primary determinant of HDF magnitude, and strong correlations with it were therefore anticipated.

The possibility that correlations are inflated by unrecognized confounders must be considered, given the limited cohort size. While HDF was strongly associated with ventricular volumes, and changed in response to a well-defined physiological perturbation—shunt-related loading and its removal by ASD closure—the modest sample size limits the ability to assess secondary modulators such as autonomic tone or myocardial contractile state.

Importantly, HDF is not a replacement for ventricular volumes. While volumetric parameters have a major impact on force magnitude, the temporal evolution and directional distribution of HDF depend on blood acceleration, chamber geometry, and ventriculo-ventricular interaction. These aspects are not captured by static volumetric indices alone and explain why correlations were direction- and phase-specific rather than uniform across all force components, as evidenced in Fig. 6 and suggested in previous work [20].

Accordingly, HDF should be viewed as a complementary descriptor of ventricular pumping mechanics rather than an independent replacement for volumetric measures. In our cohort, HDF captured changes in force orientation and timing, particularly in septal and transverse directions, even when conventional volumetric indices approached normal values after closure, suggesting sensitivity to altered flow redirection and ventricular interaction beyond EDV, SV, or EF alone. This observation suggests that HDF may have potential value for identifying incomplete functional normalization after ASD closure, a hypothesis that warrants evaluation in larger, longitudinal studies.

### 4.2 Hemodynamic forces and septal dynamics

Under normal loading conditions, the interventricular septum moves towards the LV during systole, giving a net contribution to LV function while detracting from RV pumping [34]. We have previously used HDF analysis to characterize how this septal motion pattern helps regulate the blood flow through the right ventricle [17]. As blood enters the RV through the tricuspid valve, systolic septal motion away from the RV generates a transient pressure gradient that assists in redirecting the inflowing blood around the septal curvature towards the RVOT. This motion pattern is present in all healthy hearts regardless of size [17] and is reflected in a mid-systolic hemodynamic force directed towards the septum as seen in Fig. 3, bottom left panel. By analogy to a centripetal force, the septally directed component of the hemodynamic force maintains the curvature of the intraventricular flow path.

Volume loading the right ventricle significantly alters the role of the interventricular septum, causing it to flatten during diastole, impeding LV filling [10], [34]. The subsequent systolic contraction causes the septum to regain its convex shape due to the higher systolic pressure generated by the left ventricle. This altered motion pattern results in a systolic net septal motion towards the RV, boosting RV pumping and detracting from LV pumping due to ventriculo-ventricular coupling [10]. Together, these effects significantly reduce LV stroke volumes through the Frank-Starling mechanism, impairing systemic cardiac output. To maintain adequate forward flow under such conditions, the sympathetic tone must increase, which effects increased contractility (not quantified here) and heart rate [35]. Indeed, we observed significantly lower LV stroke volumes in ASD patients before closure, concurrent with a tendency towards elevated resting heart rates, resulting in cardiac output being maintained at levels similar to controls. This compensation also serves as a reasonable explanation for the lack of difference in LV HDF when comparing patients and controls at baseline.

One of the main goals of ASD closure is to reduce the undue burden placed on the pulmonary circulation, thereby preventing the development of pulmonary hypertension. Removing the shunt normalizes biventricular filling and typically restores septal dynamics. In our study, this was manifested as a reduction in right ventricular hemodynamic forces perpendicular to the septum (Fig. 4, bottom row), restoring the previously described “curling” force responsible for redirecting blood flow from the tricuspid valve towards the right ventricular outflow tract [17]. We also noted normalization of HDF in the other two directions, reflecting the reduction in RV preload following ASD closure. These findings are partially similar to those described in an earlier study by Pola et al. of HDF in patients with precapillary pulmonary hypertension, which found elevated RV HDF in the septal-free wall direction [21]. That said, the mechanisms differ somewhat: while in ASD, the volume-loaded RV displays a diastolic septal bowing towards the LV, the pressure-loaded RV in pulmonary hypertension may exhibit septal bowing in both systole and diastole, dependent on the systolic pressure differential between the ventricles.

### 4.3 Reverse remodeling and HDF

While RV HDF decreased significantly towards normal after closure in all three directions, it remained somewhat elevated in several individuals (Fig. 4, bottom right). The remaining difference may be explained by a degree of RV dilatation and functional impairment, which may be transient or persistent, as previous longitudinal studies have described impaired RV function decades after ASD closure in childhood or adolescence [8], [36]. In a cohort largely similar to ours, right ventricular dilatation was found in 45% and systolic dysfunction (impaired RV EF, TAPSE, and/or S’) was seen in 30%–56% of subjects on echocardiography, while CMR found impaired RV EF in 6% and increased RV end-diastolic volume in 15% [8]. These findings suggest some degree of right heart dysfunction is a persisting feature in certain patients, possibly also contributing to increasing prevalence of conduction system abnormalities (QRS duration >120 ms present in 13% of patients 50 years after closure) as well as an increased arrhythmia burden [8]. Despite the increased cardiac morbidity in this group, long-term mortality remains on par with the general population. Whether HDF analysis before or soon after ASD closure is able to predict long-term RV dysfunction remains an open question.

Left ventricular forces were also affected by ASD closure, and the observed changes were only partially explained by the altered volume loading conditions. As hemodynamic forces reflect pressure gradients within the heart, which are in turn influenced by inotropic and lusitropic states, we therefore speculate that sympathetic tone contributes significantly to the observed HDF patterns, consistent with prior findings linking autonomic activity to intraventricular kinetic energy [37]. In the present study, we did not obtain any metrics allowing us to reliably assess the state of the autonomous nervous system, although some indirect indices were present, as discussed above.

### 4.4 Hemodynamic forces and absolute pressure levels

The HDF analysis framework computes global pressure gradients from the intracardiac velocity field by evaluating three terms: temporal acceleration (the local time derivative of velocity), convective acceleration, and viscous forces. The temporal acceleration term typically dominates under physiological flow conditions. Notably, because the absolute pressure levels are not part of the HDF calculation, the method reflects only temporally varying pressure gradients within the cardiac chambers. It is these dynamic pressure gradients, rather than the absolute pressures, that drive both cardiac emptying and filling [38], [39], [40]. This distinction is particularly relevant in ASD, which represents a volume overload condition without primary elevation of ventricular pressures. In this setting, substantial changes in HDF can occur despite normal or near-normal absolute pressures, reflecting altered ventricular loading, geometry, and intraventricular flow redirection rather than pressure load. Accordingly, the HDF changes observed after ASD closure should be interpreted as markers of altered volume-driven flow dynamics, not changes in absolute ventricular pressure.

### 4.5 Shunt size and hemodynamic force magnitude

Although guideline recommendations often use shunt magnitude as a decision aid for ASD closure, treatment decisions in this cohort were made prior to study inclusion based on routine clinical assessment and individual discussions with patients and families. As a result, quantitative CMR analysis revealed a broad range of shunt fractions, including some around or below 1.5. While this reflects known limitations of echocardiographic shunt estimation in children, it also represents real-world clinical practice and provided a wider physiological range of ventricular loading. This broader range strengthened the analysis of how hemodynamic forces relate to ventricular volume and how force patterns change following unloading by ASD closure.

We did not observe any significant correlation between the shunt fraction and HDF before closure in either ventricle. This likely reflects HDF being expressed in absolute numbers (Newton) that scale with the absolute amount of blood being accelerated, whereas the shunt fraction represents a dimensionless ratio between the pulmonary and systemic circuits. Consequently, a small heart may exhibit lower HDF in the presence of a relatively high shunt fraction, compared to a much larger heart with a somewhat lower shunt fraction. This interpretation is supported by the observed correlation between the reduction in shunt fraction and the change in HDF after ASD closure (Fig. 6). If HDF were normalized to ventricular volume, we would expect a correlation with shunt fraction at baseline. However, such normalization introduces interpretative ambiguities, as discussed elsewhere [19], [41].

### 4.6 Clinical implications

Hemodynamic force analysis enables a detailed yet accessible assessment of the complex blood flow patterns seen in the human heart. Previous studies have suggested HDF analysis may offer incremental information over more traditional indices of cardiac function, such as ejection fraction and ECG parameters, in specific applications [20], [21], [24]. While our findings do not directly support a clinical application for HDF analysis, we speculate that near-normal HDF levels in the presence of an ASD may indicate a low therapeutic urgency. Meanwhile, ASD symptoms typically manifest most clearly during exercise, which may also unmask latent impaired ventricular function in otherwise asymptomatic individuals decades after successful ASD correction. [42] Characterization of biventricular HDF during exercise or pharmacological stress [10], [43] is therefore warranted. Whether HDF analysis can provide incremental value for ASD patients, either for therapeutic guidance in cases with borderline hemodynamic significance, or for long-term risk stratification, remains an open question.

## 5 Limitations

We did not conduct cardiopulmonary exercise testing (CPET) and were therefore unable to assess any connection between HDF and exercise capacity. Previous studies have demonstrated that ASD is associated with decreased exercise capacity, which may persist for decades after ASD closure [5], [8], [44], [45], [46]. Future studies should seek to evaluate whether baseline HDF, or postoperative HDF recovery predicts the degree and persistence of exercise capacity impairment.

A small number of patients had partial anomalous pulmonary venous return, which represents an extracardiac left-to-right shunt and a volume-loading physiology similar to ASD; any influence on the present results is therefore expected to be limited.

Our study used a small but well-matched cohort in a single-center setting. While we mitigated the small number by employing state-of-the-art measurement techniques and a paired observational design, any small study risks limited generalizability. We believe the physiological significance of the findings should translate well beyond the sampled population, however, as HDF reflects fundamental physical concepts relating to cardiac pumping and blood flow patterns.

We used a relatively short follow-up duration, as evident from the yet non-normalized RV EF at follow-up. Over time, biventricular function is expected to normalize in most subjects [8], especially those treated early in life; however, we did not aim to fully characterize the coupling between HDF and long-term functional recovery after ASD closure.

## 6 Summary

In this manuscript, we present an analysis of hemodynamic forces from 4D Flow CMR in a cohort of children and adolescents before and after undergoing atrial septal defect closure. In this cohort, hemodynamic forces were significantly altered compared to matched controls at baseline, while ASD closure normalized biventricular volumes as well as hemodynamic forces. Hemodynamic forces were only partially explained by cardiac volumetry, suggesting they are influenced by factors beyond preload, such as inotropic and lusitropic modulation. Whether hemodynamic forces may provide incremental information of prognostic or therapeutic significance remains unknown.

## Funding

P.A., H.C., and P.S. were supported by Region Skåne (2022–0288, 2022–1460, 2025–2749). P.A. and P.S. were supported by grants from The Swedish Heart-Lung Foundation (20241381, 20240862, 20240935, 20220369), Maggie Stephens Foundation, and Lund University Faculty of Medicine. P.A. was also supported by Bundy Academy. The remaining authors declare no conflicts of interest, financial or otherwise.

## Author contributions

**Per M Arvidsson:** Writing – review & editing, Writing – original draft, Visualization, Resources, Methodology, Investigation, Funding acquisition, Formal analysis, Conceptualization. **Henning Clausen:** Writing – review & editing, Project administration, Investigation, Formal analysis, Data curation. **Pia Sjöberg:** Writing – review & editing, Supervision, Resources, Investigation, Funding acquisition, Data curation.

## Declaration of competing interests

The authors declare that they have no known competing financial interests or personal relationships that could have appeared to influence the work reported in this paper.

## Declaration of Generative AI and AI-assisted technologies in the writing process

During the preparation of this work, the authors used ChatGPT (OpenAI, San Francisco, California) for language editing and stylistic refinement of the manuscript text. After using this tool, the authors carefully reviewed and revised all generated content and take full responsibility for the final version of the article.
