# Supplementary material for: Closure of atrial septal defect normalizes left and right ventricular hemodynamic forces in children and adolescents
Source: J Cardiovasc Magn Reson. 2026 Feb 12;28(1):102704. doi: 10.1016/j.jocmr.2026.102704 (PMC13213871; doi:10.1016/j.jocmr.2026.102704)
Supplement: Supplementary file 1 — Supplementary material [file mmc1.docx]

**
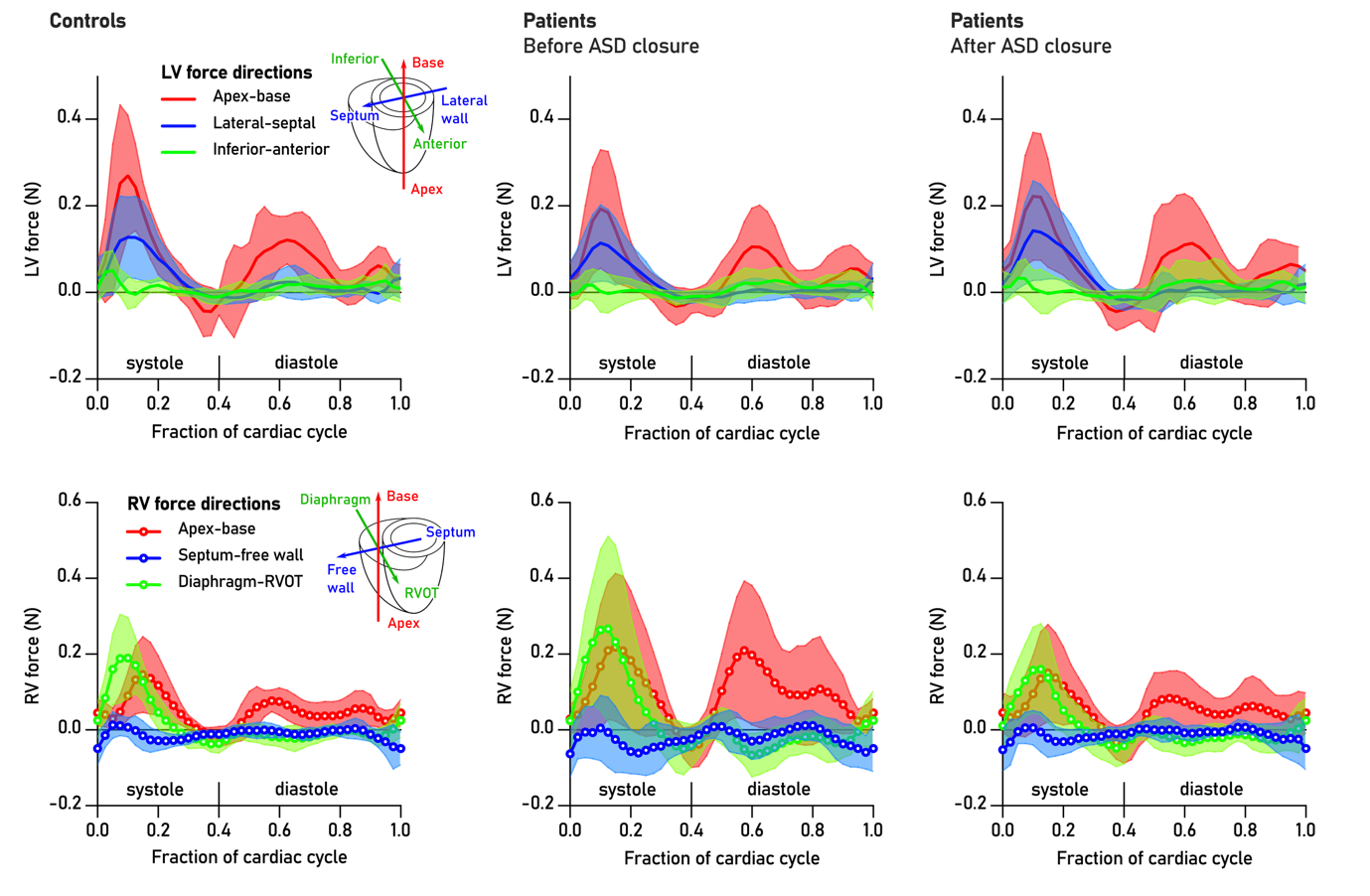
**

**Supplemental Figure 1.** Biventricular HDF curves for controls (left column), patients before ASD closure (center), and patients after closure (right). Mean values ±1SD. The averaged curves qualitatively reproduce the temporal patterns evident in the individual examples shown in Figure 3 and confirm that the observed changes are not driven by isolated subjects. We have not included these averaged curves in the manuscript, as the cohort spans a wide age range (4–17 years) with large inter-individual differences in cardiac size and absolute force magnitude. The averages of absolute forces are not intended to imply a degree of physiological homogeneity.
